# Relationship between social capital and quality of life among adult stroke patients: a cross-sectional study in Anhui Province, China

**DOI:** 10.1186/s12955-022-01925-x

**Published:** 2022-02-05

**Authors:** Kai Ji, Zhongliang Bai, Yan Zhao, Lingzhi Sang, Danni Wang, Ren Chen

**Affiliations:** 1 School of Health Services Management, Anhui Medical University, Hefei, 230032 China; 2 Anhui Maternal and Child Health Hospital, Hefei, 230001 China; 3 School of Public Health, Anhui Medical University, Hefei, 230032 China; 4 Suzhou Hospital, Anhui Medical University, Suzhou, 234000 Anhui China

**Keywords:** Social capital, Quality of life, Stroke, Health management, Social medicine, Cross-sectional study

## Abstract

**Objectives:**

Few studies have investigated the association between social capital and quality of life (QoL) among stroke patients. To address this research gap, we aimed to explore the association between social capital and QoL among stroke patients in Anhui Province, China.

**Study design:**

Cross-sectional study.

**Methods:**

This cross-sectional study was conducted using a multi-stage stratified random sampling method. The following data including demographic characteristics, health-related conditions, five dimensions of social capital status, and quality of life (QoL) were collected using a questionnaire. Generalized linear models were then used to determine the relationship between social capital and QoL after adjusting for confounding factors.

**Results:**

A total of 390 participants were included for the final analysis in this study. Our results indicated that subjects with higher social capital including social connection (coefficient: 28.28, 95% CI: 19.39–37.16), social support (coefficient: 21.17, 95% CI: 10.63–31.71), trust (coefficient: 13.46, 95% CI: 2.73–24.19), reciprocity (coefficient: 25.56, 95% CI: 15.97–35.15), and cohesion (coefficient: 19.30, 95% CI: 9.90–28.70) had increased odds of reporting poor QoL when compared with lower social capital group. We also observed that the association between social capital and QoL varied across cities.

**Conclusions:**

Our findings show that social capital is associated with QoL in adult stroke patients, suggesting that social capital may be significant for enhancing QoL among adults with stroke.

**Supplementary Information:**

The online version contains supplementary material available at 10.1186/s12955-022-01925-x.

## Introduction

Stroke is characterized by high morbidity, disability, mortality, and recurrence, and is the primary cause of death and disability for adult men and women worldwide [1]. The 2016 Global Burden of Disease data shows that stroke is the leading cause of years of life lost in China [2], which indicates that stroke is a great challenge. While the incidence and prevalence of stroke have continued to rise in recent years [3, 4]. In addition, many patients encounter anxiety, depression, and interpersonal conflicts, which negatively influence their daily existence and quality of life (QoL) [5, 6]. Previous studies have reported that good QoL is vital for the health and well-being of adults, especially stroke patients [7]. Therefore, how to improve the QoL of these people deserves more attention. Several studies have shown that younger age, normal weight, educational attainment, physical activity, and a higher income can improve QoL [8–10]. Besides, with the development of social determinants, the role of social factors on the QoL of stroke patients has been well recognized, especially their social relations or mutual connections. In practice, patients with chronic diseases like stroke may suffer from physical and mental challenges, which limits the growth and development of social networks to obtain material and emotional social support, in turn, compromising the QoL [11]. Therefore, special attention should be paid to the social relations or mutual connections among stroke patients.

Social capital, as an individual’s social network, which comprises several dimensions with each dimension being used to describe a phenomenon concerning social relations at individual and societal levels [12–16]. Currently, social capital has been recognized as a social determinant and health-impacting factor [14, 17, 18], which also is an important factor in chronic disease prevention and control [19–21]. Studies on the relationship between social capital including social participation and social support, and stroke patients have reported that social capital has a positive role in the prevention and treatment of strokes [22–25]. In other words, a comprehensive appreciation of social capital associated with QoL is of great importance to devise appropriate and specific strategies aimed to improve prognosis and well-being among these communities. However, previous studies that look into the association between social capital and quality of life among stroke patients have mostly come from developed or western countries. While such study is scarce in China, the largest developing country. For example, a study from Saudi Arabia found that social support positively influences the QoL among stroke survivors [26]. While a prospective cohort study in German concluded that having a small social network was negatively associated with QoL [27]. To contribute to existing findings, we aimed to address this research gap by exploring the association between social capital and QoL among stroke patients in Anhui Province, China.

## Methods

### Study design and data collection

To explore the relationship between social capital and QoL among stroke patients, we conducted this cross-sectional survey in Anhui Province, eastern China, between August and October 2014. This study was approved by the Biomedical Ethics Committee of our university (No.20150297).

Moreover, we used a multi-stage stratified random sampling method considering socio-economic levels and geographic location in order to ensure a representative sample. Therefore, we selected three cities in Anhui Province: Fuyang (lower socio-economic level; north); Tongling (middle socio-economic level; south); and Hefei (higher socio-economic level; central) [28] (Additional file 1).

Next, in these selected cities, we selected township or community healthcare centers by a random lottery, and then we randomly identified potential participants (random number according to the healthcare record number). Individuals diagnosed with stroke in secondary hospitals and above, using information obtained from the chronic disease database of the local Center for Disease Control and Prevention, which included all potential participants, were identified as our participants. The sample size was evaluated by the following formula:

$$N={{Z}^{2}}_{\alpha /2}\left(1-\mathrm{P}\right)/{\varepsilon }^{2}\mathrm{P}=385,\mathrm{ \alpha }=0.05,\upvarepsilon =0.10, {{ Z}^{2}}_{\alpha /2}=1.96, P=0.5.$$

With assistance from local community workers, skilled or trained graduate students personally conducted structured face-to-face interviews with participants at the Community Center. The investigators verbally explained the purposes and procedures of the study to the interviewees who were then requested to fill out voluntary consent forms before the interviews began.

## Measures

### Quality of life

To determine the QoL in stroke patients, we used a 59-item simplified Chinese translated version of the Stroke Impact Scale (SIS) questionnaire, which was developed by Duncan and been validated in China previously [29]. The SIS is comprised of eight dimensions: strength, hand functioning, activities of daily living, instrumental activity of daily living (ADL/IADL), locomotive function, communication, emotion, memory, and participation. Response options range from 1 to 5 on a Likert scale and the QoL scores are calculated by summing all 59 items. It is worth noting that higher scores represent better QoL. In this study, the overall Cronbach’s α coefficient of the SIS was 0.98. Moreover, the Cronbach’s α coefficients of the eight dimensions ranged from 0.83 to 0.99, suggesting excellent reliability in our study sample.

### Social capital

The Integrated Questionnaire for the Measurement of Social Capital (SC-IQ) developed by World Bank, including six domains regarding groups and networks, trust and solidarity, collective action, and cooperation, information and communication, social cohesion and inclusion, and empowerment and political action [30]. Based on the World Bank’s SC-IQ and previous works [11, 31, 32], social capital (including five dimensions, such as social support, social connection, trust, cohesion, and reciprocity) was included in the present study. We selected 21 commonly used and easily understood items to measure social capital and adapted them to the Chinese context (Additional file 2). More details about the assessment of social capital have been fully described in previously published papers from our research group [11, 12, 32–34]. In this study, we adopted a five-point Likert scale for the social capital questionnaire where respondents were asked to rate their agreement (1 = “never,” 2 = “seldom,” 3 = “usually,” 4 = “often,” and 5 = “more often”). For each social capital domain, answers to varied items were summarized in order to obtain an overall score. Higher scores indicated a better social capital status. While analyzing the data, we dichotomized the scores of each social capital dimension by taking the median value as the cut-off [35, 36] including social support (high [≥ 12] and low [< 12]), social connection (high [≥ 15] and low [< 15]), trust (high [≥ 20] and low [< 20]), cohesion (high [≥ 14] and low [< 14]), and reciprocity (high [≥ 14] and low [< 14]). The overall Cronbach’s α coefficient was 0.88 for the scale, 0.68 for the social connection dimension, 0.69 for the social support dimension, and over 0.8 for the other dimensions, respectively.

### Other variables

We also collected information on demographic and health-related variables. Basic demographic variables included age (41–50, 51–60, 61–70, 71–80, and > 80 years), gender (male, female), body mass index (BMI) (< 24.0, 24.0–26.9, 27.0–29.9, and ≥ 30.0 kg/m^2^), living status (living alone or with others), marital status (married/cohabiting, and single including never married, divorced, and widowed), and education (primary school and below, junior high school, high school, and above). We also collected data on health-related variables such as blood pressure, exercising status, drinking status, smoking status, stroke type, time since stroke onset, medication, and multimorbidity. We asked participants whether they had been diagnosed with hypertension, coronary heart disease, benign tumors, malignant tumors, chronic obstructive pulmonary disease, diabetes (type 1 or 2), or neurological disorders. Finally, participants were categorized into a multimorbidity group if they had at least one of the above-mentioned diseases.

### Statistical analysis

Continuous variables are presented as mean ± standard deviation, while categorical variables are presented as percentages (%). Firstly, a one-way analysis of variance (ANOVA) was used to compare the differences in the total quality of life scores in each group, which are described by different demographic characteristics. Next, a generalized linear model (GLM) was used to investigate the relationship between different social capital dimensions and QoL scores. The GLM model can be specified as follows:

$$QoL scores \approx \alpha +{\beta }_{1}Social capital dimensions +{\beta }_{2}{Confounders}_{1}+\dots +{\beta }_{n}{Confounders}_{n}$$

where QoL score is the dependent variable; $\alpha$ is the intercept; social capital dimensions refer to the above-mentioned five dimensions of social capital; ${\beta }_{1}$ is the corresponding coefficient; and ${Confounders}_{1}+\dots +{\beta }_{n}{Confounders}_{n}$ indicates potential confounders in the model and their corresponding coefficients $({\beta }_{2}\dots {\beta }_{n})$. According to previous studies which found that age, exercising status, drinking status, smoking status, stroke type were linked to the quality of life of stroke patients [37–40], we took these variables as potential confounders in the generalized linear model. Moreover, the GLM model was stratified according to the socio-economic levels used for analysis. All statistical analyses were performed using IBM SPSS 23.0 statistical software and *P* < 0.05 was considered statistically significant.

## Results

### Descriptive analysis

A total of 420 stroke patients were identified, of which 18 participants were excluded due to those who could not fully understand the verbal explanations due to severe deafness and limited communication skills. Then, 12 participants were excluded due to missing values. Finally, 390 of 420 (92.86%) stroke patients were included during data analysis. The characteristics of the participants included: BMI < 24.0 (57.18%), living with others (86.41%), married or cohabited (76.41%), attended primary school and below (72.82%), SBP < 140 (52.82%), DBP < 90 (62.31%), exercise three or more times per week (47.18%), non-drinking (66.67%), non-smoking (63.33%), ischemic stroke (85.64%), more than 24 months since stroke onset (64.10%), taking medicine (86.67%), and non-multimorbidity (57.18%) (Table 1). The QoL scores of most of these groups were distributed between 202 and 231. Results revealed statistical differences regarding the type of stroke, medication, age, smoking, drinking, and exercising status, (*P* < 0.05).

**Table 1:** Descriptive results of participants characteristics (N = 390)

Variables	Total n = 390	Quality of life score	*P-*value
*Age, years, N (%)*			0.034
41–50	9 (2.31)	216.56 ± 53.64	
51–60	42(10.70)	217.81 ± 45.58	
61–70	167(42.82)	224.20 ± 44.66	
71–80	132(33.85)	215.88 ± 48.32	
> 80	40 (10.26)	189.03 ± 64.00	
*Gender, N (%)*			0.507
Male	199(51.03)	218.54 ± 51.10	
Female	191(48.97)	215.22 ± 47.41	
*BMI, kg/m*^*2*^*, N(%)*			0.191
< 24.0	223(57.18)	213.25 ± 52.49	
24.0–26.9	98 (25.13)	223.46 ± 46.29	
27.0–29.9	41 (10.51)	225.68 ± 43.09	
≥ 30.0	28 (7.18)	210.36 ± 38.37	
*Living status, N(%)*			0.132
Living alone	53 (13.59)	213.15 ± 36.50	
Living with others	337(86.41)	217.50 ± 51.03	
*Marital status, N(%)*			0.768
Married/cohabited	298(76.41)	217.32 ± 50.47	
Single	92 (23.59)	215.59 ± 45.49	
*Education, N(%)*			0.058
Primary school and below	284(72.82)	213.31 ± 48.05	
Junior high school	67 (17.18)	225.39 ± 49.29	
High school and above	39 (10.00)	228.56 ± 55.60	
*Blood pressure (SBP), N(%)*			0.538
< 140	206(52.82)	218.37 ± 47.19	
≥ 140	184(47.18)	215.28 ± 51.61	
*Blood pressure (DBP), N (%)*			0.987
< 90	243(62.31)	217.92 ± 46.23	
≥ 90	147(37.69)	215.25 ± 54.09	
*Exercising status, N (%)*			< 0.001
Never	175(44.87)	204.54 ± 53.45	
1–2times a week	31 (7.95)	220.32 ± 35.02	
3times a week and above	184(47.18)	228.10 ± 44.41	
*Drinking status, N(%)*			< 0.001
Current drinking	40 (10.26)	245.90 ± 25.99	
Former drinking	90 (23.08)	213.07 ± 49.67	
Nondrinking	260(66.67)	213.78 ± 50.54	
*Smoking status, N(%)*			0.019
Current smoking	66 (16.92)	233.23 ± 38.57	
Former smoking	77 (19.74)	213.70 ± 49.60	
Nonsmoking	247(63.33)	213.55 ± 51.01	
*Stroke type, N(%)*			< 0.001
Hemorrhagic stroke	56 (14.36)	204.41 ± 53.76	
Ischemic stroke	334(85.64)	219.01 ± 48.27	
*Time since stroke onset, months, N(%)*			
< 6	54 (13.85)	214.57 ± 51.43	0.387
6–12	28 (7.18)	204.93 ± 53.90	
13–24	58 (14.87)	224.17 ± 49.45	
> 24	250(64.10)	217.08 ± 48.25	
*Medication, N(%)*			0.005
Yes	338(86.67)	214.14 ± 49.60	
No	52 (13.33)	234.92 ± 43.53	
*Multimorbidity*			0.355
Yes	167(42.82)	214.24 ± 48.78	
No	223(57.18)	218.91 ± 49.68	

Continuous variables are presented as mean ± standard deviation, categorical variables are presented as number (%)

*P-*Value: derived from the variance analysis

Table 2 shows that the group with high social connection, social support, trust, reciprocity, and cohesion accounted for 51.28%, 74.62%, 74.62%, 65.38%, and 62.31%, respectively. Moreover, the analysis showed that QoL scores among stroke patients were significantly different in all five dimensions of social capital.

**Table 2:** Descriptive results of social capital (N = 390)

Variables	Total n = 390	QoL scores	*P-*value
*Social connection*			< 0.001
Low	190(48.72)	201.3 ± 50.13	
High	200(51.28)	231.75 ± 43.68	
*Social support*			< 0.001
Low	99(25.38)	200.45 ± 47.97	
High	291(74.62)	222.51 ± 48.55	
*Trust*			0.002
Low	99(25.38)	203.98 ± 49.83	
High	291(74.62)	221.31 ± 48.4	
*Reciprocity*			< 0.001
Low	135(34.62)	197.67 ± 50.82	
High	255(65.38)	227.1 ± 45.35	
*Cohesion*			< 0.001
Low	147(37.69)	204.24 ± 49.3	
High	243(62.31)	224.58 ± 47.77	

Continuous variables are presented as mean ± standard deviation, categorical variables are presented as number (%)

*P-*Value: derived from the variance analysis

### Generalized linear model

Table 3 shows the GLM results. After controlling for confounders (including stroke type, medication, age, exercising, drinking, and smoking status), effects of the five dimensions of social capital became attenuated but were positively associated with quality of life. In the total population, the QoL score of the higher social capital group increased by 28.28, 21.17, 13.46, 25.56, and 19.30, respectively, in each dimension when compared with the lower social capital group.

**Table 3:** The relationship between social capital and QoL using GLM (N = 390)

Social capital dimensions	Unadjusted			Adjusted		
	B(S.E.)	95% CI	*P*-Value	B(S.E.)	95% CI	*P*-Value
*Overall*						
Social connection						
High	30.45(4.75)	21.10, 39.79	< 0.001	28.28(4.52)	19.39, 37.16	< 0.001
Low	Reference			Reference		
Social support						
High	22.06(5.63)	10.98, 33.13	< 0.001	21.17(5.36)	10.63, 31.71	< 0.001
Low	Reference			Reference		
Trust						
High	17.33(5.67)	6.18, 28.49	0.002	13.46(5.46)	2.73, 24.19	0.014
Low	Reference			Reference		
Reciprocity						
High	29.42(5.04)	19.52, 39.33	< 0.001	25.56(4.88)	15.97, 35.15	< 0.001
Low	Reference			Reference		
Cohesion						
High	20.34(5.05)	10.41, 30.28	< 0.001	19.30(4.78)	9.90, 28.7	< 0.001
Low	Reference			Reference		
*Fuyang*						
Social connection						
High	43.12(6.84)	29.6, 56.65	< 0.001	39.46(6.6)	26.4, 52.53	< 0.001
Low	Reference			Reference		
Social support						
High	29.34(8.48)	12.57, 46.12	0.001	28.71(8.09)	12.72, 44.71	0.001
Low	Reference			Reference		
Trust						
High	15.36(8.53)	− 1.50, 32.23	0.074	12.43(8.34)	− 4.06, 28.92	0.138
Low	Reference			Reference		
Reciprocity						
High	36.67(7.67)	21.5, 51.83	< 0.001	36.59(7.36)	22.02, 51.16	< 0.001
Low	Reference			Reference		
Cohesion						
High	23.01(7.59)	8.01, 38.01	0.003	19.14(7.46)	4.40, 33.89	0.011
Low	Reference			Reference		
*Tonling*						
Social connection						
High	21.07(10.26)	0.76, 41.38	0.042	14.64(9.61)	− 4.39, 33.67	0.130
Low	Reference			Reference		
Social support						
High	11.34(12.71)	− 13.81, 36.48	0.374	2.02(12.08)	− 21.91, 25.94	0.868
Low	Reference			Reference		
Trust						
High	25.15(14.79)	− 4.12, 54.42	0.092	8.96(13.85)	− 18.46, 36.38	0.519
Low	Reference			Reference		
Reciprocity						
High	26.52(10.89)	4.97, 48.07	0.016	17.02(10.31)	− 3.39, 37.44	0.101
Low	Reference			Reference		
Cohesion						
High	33.06(11.29)	10.72, 55.40	0.004	28.73(10.47)	8.01, 49.45	0.007
Low	Reference			Reference		
*Hefei*						
Social connection						
High	26.67(7.3)	12.23, 41.11	< 0.001	26.51(6.99)	12.66, 40.36	< 0.001
Low	Reference			Reference		
Social support						
High	24.83(8.16)	8.68, 40.98	0.003	26.03(7.82)	10.55, 41.51	0.001
Low	Reference			Reference		
Trust						
High	19.24(7.93)	3.55, 34.93	0.017	15.68(7.96)	− 0.09, 31.45	0.051
Low	Reference			Reference		
Reciprocity						
High	25.64(7.51)	10.78, 40.50	0.001	22.03(7.55)	7.07, 36.98	0.004
Low	Reference			Reference		
Cohesion						
High	10.35(7.73)	− 4.95, 25.65	0.183	10.14(7.49)	− 4.70, 24.98	0.178
Low	Reference			Reference		

Adjusted by Age, smoking status, drinking status, exercising status, type of stroke, and medication

B: regression coefficient

S.E.: standard error

95% CI: confidence interval of 95%

However, the association between social capital dimensions and QoL scores was different in cities with different socio-economic levels. In Fuyang (lower socio-economic level), all dimensions were statistically associated with QoL scores, except for trust. However, only cohesion was positively associated with QoL scores in Tongling (middle socio-economic level). Furthermore, social connection, social support, trust, and reciprocity were not statistically associated with QoL scores. In Hefei (higher socio-economic level), social connection, social support, and reciprocity were positively associated with QoL scores, while trust and cohesion were not statistically significant.

## Discussion

This study was to examine the relationship between social capital and QoL among stroke patients in China. Our results found a positive association between social capital and QoL. In other words, patients with a higher level of social connection, social support, trust, reciprocity, and cohesion had better QoL, which added the findings to existing studies. Furthermore, the stratified analysis results indicated that such findings persisted after separation using different socio-economic level areas.

Previous studies have shown that there was a positive relationship between social capital and QoL among different communities including older adults [41–43], women [44, 45], children [46], and patients with fibromyalgia, multiple sclerosis, and HIV/AIDS [12, 47, 48]. Likely, findings of this study documented that some dimensions of social capital were linked to the QoL among stroke patients. Specifically, we found that stroke patients with a higher level of social connection had higher QoL than those with a lower level of social connection. This finding is in line with a previous study from the United States [49], which suggests that stroke patients with a higher social connection are healthier, are more optimistic rather than negative, and have frequent social contacts and interactions that offer support and care, thereby improving QoL [50, 51]. In other words, a higher degree of social interaction could help stroke patients gain more knowledge, thus, benefits the process of rehabilitation and in turn, improving the QoL.

Our results from the adjusted GLM indicated a significant positive association between social capital with regard to social support, trust, and QoL. Similarly, previous research reported that social support is an important factor in stroke patients from Malawi [52]. Meanwhile, another study in 25 European Countries investigated the relationship between trust and mortality found that increasing institutional trust prompts a reduction in COVID-19 mortality [53]. Moreover, a previous study showed that stroke patients who had trusting relationships with their family members and medical workers manifested an optimistic mood and behavior, which encouraged independence, coping with the disease, and achieved positive results in therapy [54]. A study also showed that socially isolated stroke patients are more likely to have a recurrent stroke and have higher mortality [55]. Therefore, good social support and trust systems have a positive role on the health of stroke patients, possible mechanism may be that social support and trust encourage mutual respect and support in social interaction, thereby making it easier to gain self-satisfaction and reflect self-worth.

In addition, adequate mental and financial support from family members makes it easier for patients to believe that they are cared for and loved, thus, enabling a sense of security which improves prognosis and QoL [26, 56]. Furthermore, interpersonal trust increases a sense of security and encourages stroke patients to participate in social activities, especially to build trust with family, friends, neighbors, and community doctors [57, 58]. This helps patients actively cooperate with rehabilitation after illness [59], which is good for their health and improves QoL.

Results also indicated that a higher level of reciprocity was associated with better QoL among stroke patients, which is consistent with a previous finding that neighborhood social reciprocity influenced the mental health of older adults in China [60]. The possible explanation may be those stroke patients who are more willing to help others and a better mental state in return commonly.

We also found a positive correlation between QoL and cohesion. Similarly, Chen et al*.* [61] reported that social cohesion and trust was important variables that influenced self-rated health and happiness. Cohesion, a component of cognitive social capital, plays an important role in increasing mental wellbeing such as individual security and self-worth through psychosocial mechanisms [62]. Therefore, a higher level of cohesion among stroke patients was able to promote the individual to preserve mental health, ultimately improving QoL.

Moreover, our results indicated that certain dimensions of social capital among stroke patients were associated with QoL at different socio-economic level cities. In particular, social connection, social support, reciprocity, and cohesion were statistically correlated with QoL among stroke patients from a lower socio-economic level area. Only cohesion was associated with QoL among stroke patients from a middle socio-economic level area. Finally, social connection, social support, and reciprocity were significant for stroke patients from an area with a higher socio-economic level. Our study also indicated that some factors of a region including socioeconomic levels could influence the building and construction of social capital [63], and pathways that connect social capital with health may vary in different economic settings [64]. Consequently, economic levels should be well considered when linking social capital to health outcomes [65].

Our results indicated a mixed pattern of relationships which revealed that certain dimensions of social capital play a role across the socio-economic level and highlighted its function in constructing and building social capital [63, 64]. Firstly, this finding suggests that social capital should be included when taking measures to enhance QoL among stroke patients. Secondly, it adds to previously reported results on the disparity in social capital in areas of different socio-economic levels. In addition, it reveals the necessity to assess social capital using several dimensions instead of just one dimension [12].

In view of the findings of this paper, to improve the quality of life among stroke patients, some useful programs or initiatives measures to build and strengthen social capital concerning social networks, social support, trust, reciprocity, and cohesion from family members, relatives, neighborhoods, and friends should be introduced. First, earlier studies found that stroke patients lacked communication with others and confronted a sense of loneliness from time to time [66, 67]. Thereby, this study put forward that different kinds of support systems consisting of mental or material support should be developed and the relatives or family members, friends, neighbors of the patients are encouraged to be more frequent contact with them. Besides, programs intended to cultivate and escalate the awareness of community cohesion should also be introduced. Moreover, different socioeconomic factors should be well considered when taking advantage of the above-mentioned programs or initiatives. Second, existing models of stroke patient care are relatively simple, resulting in greater pressure often caregivers, their health is also affected [68]. Therefore, we suggest that the care model of the stroke patients should be re-organized and coordinated with the help of the whole community members, including local CDC, community health service staff, clinical staff, volunteers, family members, relatives, friends, and colleagues, etc. Lastly, in public health practice, findings from our paper can not only shed light on how to improve the quality of life among stroke patients from the perspective of social capital but also facilitate the development of social capital theory, which can inform the policymakers of the relevance and importance of social capital.

Several limitations should be acknowledged. First, causal relations between social capital and QoL could not be verified because it was a cross-sectional study. Therefore, further studies using a longitudinal or randomized control trial design should be conducted. Second, the generalization of our findings is limited since the study was only conducted in Anhui Province. Future studies that include expanded areas and larger samples are needed. Third, data in the present study were based on self-report and might be subject to a recall or reporting bias. Nevertheless, we formulated clear and precise questions and carried out a pilot study before the investigation to improve the data accuracy. During data collection, forward or backward recall techniques were also used. Fourth, the data used in this paper was obtained from 2014, which might comprise its relevance for today. However, stroke is still a challenging public health issue in China even today, and given that its stable epidemic characteristics in recent years [69], some findings obtained from the current study still could be of relevance for today. Moreover, some results analyzed from this database had been published previously [11]. The data are not recent and therefore may not represent the more recent estimates of social capital and QoL. However, the data provides the opportunity to investigate a research topic that has not been studied in a Chinese setting before. The findings are expected to be useful in the management and health promotion of stroke patients and to be of interest among researchers in the areas of physical and mental health in China.

## Conclusion

In summary, we observed an association between social capital and quality of life among adults with stroke in Anhui Province. In particular, a high level of social connection, social support, trust, reciprocity, and cohesion improved the quality of life of stroke patients. Therefore, targeted attention should be paid to stroke patients from regions with different economic levels. Our findings will help in developing strategies to enhance treatment, nursing, and rehabilitation services, and lessen the associated negative impacts on individuals, families, and society, thereby enhancing the QoL of stroke patients.

## Supplementary Information

**Additional file 1:** The location of sampling areas in Anhui province, China.

**Additional file 2:** The Questionnaire of this study (English version).

## Data Availability

The datasets analysed during the current study are available from the corresponding author on reasonable request.
