# Supplementary figures and images for: Relationship between social capital and quality of life among adult stroke patients: a cross-sectional study in Anhui Province, China
Source: Health Qual Life Outcomes. 2022 Feb 5;20:19. doi: 10.1186/s12955-022-01925-x (PMC8817153; doi:10.1186/s12955-022-01925-x)

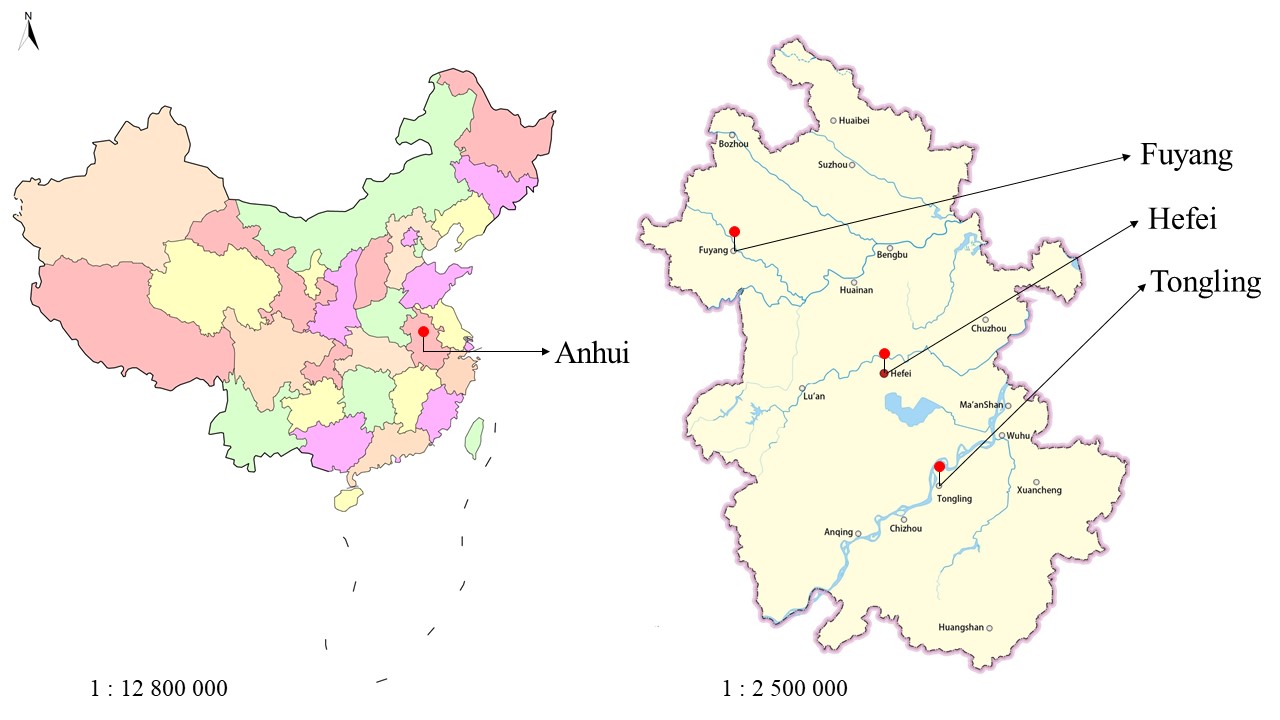

Supplement: Supplementary file 1 — Additional file 1: The location of sampling areas in Anhui province, China. [file 12955_2022_1925_MOESM1_ESM.docx]
