# Supplementary material for: Relationship between social capital and quality of life among adult stroke patients: a cross-sectional study in Anhui Province, China
Source: Health Qual Life Outcomes. 2022 Feb 5;20:19. doi: 10.1186/s12955-022-01925-x (PMC8817153; doi:10.1186/s12955-022-01925-x)
Supplement: Supplementary file 2 — Additional file 2: The Questionnaire of this study (English version). [file 12955_2022_1925_MOESM2_ESM.docx]

| *1.Age* | | | | | | |
| --- | --- | --- | --- | --- | --- | --- |
| *2.Gender* | | | | | | |
| Male | | | | Female | | |
| *3.Height (cm)* | | | | | | |
| *4.Weight (kg)* | | | | | | |
| *5.Living status* | | | | | | |
| Living alone | | | | Living with others | | |
| *6.Marital status* | | | | | | |
| Married /cohabited | | | | Single | | |
| *7.Education level* | | | | | | |
| Primary school and below | | | Junior high school | | | High school and above |
| *8.Systolic blood pressure(mmHg)* | | | | | | |
| *9.Diastolic blood pressure(mmHg)* | | | | | | |
| *10.Exercising status* | | | | | | |
| Never | | 1-2times a week | | | 3times a week and above | |
| *11.Drinking status* | | | | | | |
| Current drinking | | Former drinking | | | Nondrinking | |
| *12.Smoking status* | | | | | | |
| Current smoking | | Former smoking | | | Nonsmoking | |
| *13.Stroke type* | | | | | | |
| Hemorrhagic stroke | | | | Ischemic stroke | | |
| *14.Time since stroke onset(months)* | | | | | | |
| *15.Are you taking drugs for stroke?* | | | | | | |
| Yes | | | | No | | |
| *16.Have you been diagnosed with the following chronic diseases?* | | | | | | |
| Hypertension | Coronary heart disease | | | | Benign tumors | |
| Malignant tumors | Neurological disorders | | | | Diabetes (type 1 or 2) | |
| Chronic obstructive pulmonary disease | | | | | | |

***Demography and Basic Health Questionnaire***

| **Social Capital** | | | | | | |
| --- | --- | --- | --- | --- | --- | --- |
| Dimension | Questions | Never | Seldom | Usually | Often | More often |
| Social Support | When you are in trouble, is there someone that provides you with mental support (i.e., comfort you)? | 1 | 2 | 3 | 4 | 5 |
|  | When you are in trouble, is there someone that provides you with material support (i.e., lend you money)? | 1 | 2 | 3 | 4 | 5 |
|  | When you are in trouble, are there any formal or informal groups that provide you with mental support (i.e., comfort you)? | 1 | 2 | 3 | 4 | 5 |
|  | When you are in trouble, are there any formal or informal groups that provide you with material support (i.e., lend you money)? | 1 | 2 | 3 | 4 | 5 |
| Social Connection | How often do you contact with your relatives or family members? | 1 | 2 | 3 | 4 | 5 |
|  | How often do you contact with your friends? | 1 | 2 | 3 | 4 | 5 |
|  | Do you know the neighbors around? | 1 | 2 | 3 | 4 | 5 |
|  | How often do you contact with your neighbors? | 1 | 2 | 3 | 4 | 5 |
| Trust | Do you trust in your relatives or family members? | 1 | 2 | 3 | 4 | 5 |
|  | Do you trust in your friends? | 1 | 2 | 3 | 4 | 5 |
|  | Do you trust in your neighbors? | 1 | 2 | 3 | 4 | 5 |
|  | Do you trust in community/village doctor? | 1 | 2 | 3 | 4 | 5 |
|  | Do you trust in staff of community/village committee? | 1 | 2 | 3 | 4 | 5 |
| Cohesion | Do you think people have a good relationship here? | 1 | 2 | 3 | 4 | 5 |
|  | Do you care about your community/village? | 1 | 2 | 3 | 4 | 5 |
|  | Do you think other people care about this community/village? | 1 | 2 | 3 | 4 | 5 |
|  | Do you feel reluctant, if you have to move away from the community lived now? | 1 | 2 | 3 | 4 | 5 |
| Reciprocity | When your family members or relatives are in trouble, will you provide help to them? | 1 | 2 | 3 | 4 | 5 |
|  | When your neighbors are in trouble, will you provide help to them? | 1 | 2 | 3 | 4 | 5 |
|  | When your friends are in trouble, will you provide help to them? | 1 | 2 | 3 | 4 | 5 |
|  | When some strangers are in trouble, will you provide help to them? | 1 | 2 | 3 | 4 | 5 |

| **Stroke Impact Scale** | | | | | | |
| --- | --- | --- | --- | --- | --- | --- |
| The purpose of this questionnaire is to evaluate how stroke has impacted your health and life. We want to know from YOUR POINT OF VIEW how stroke has affected you. We will ask you questions about impairments and disabilities caused by your stroke, as well as how stroke has affected your quality of life. Finally, we will ask you to rate how much you think you have recovered from your stroke. | | | | | | |
| These questions are about the physical problems that may have occurred as a result of your stroke. | | | | | | |
| 1. In the past week, how would you rate the strength of your … | A lot of strength | | Quite a bit of strength | Some strength | A little strength | No strength at all |
| a. Arm that was most affected by your stroke? | 5 | | 4 | 3 | 2 | 1 |
| b. Grip of your hand that was most affected by your stroke? | 5 | | 4 | 3 | 2 | 1 |
| c. Leg that was most affected by your stroke? | 5 | | 4 | 3 | 2 | 1 |
| d. Foot/ankle that was most affected by your stroke? | 5 | | 4 | 3 | 2 | 1 |
| These questions are about your memory and thinking. | | | | | | |
| 2. In the past week, how difficult was it to … | | Not difficult at all | A little difficult | Somewhat difficult | Very difficult | Extremely difficult |
| a. Remember things that people just told you? | | 5 | 4 | 3 | 2 | 1 |
| b. Remember things that happened yesterday? | | 5 | 4 | 3 | 2 | 1 |
| c. Remember to do things (eg, keep scheduled appointments or take medication)? | | 5 | 4 | 3 | 2 | 1 |
| d. Remember the day of the week? | | 5 | 4 | 3 | 2 | 1 |
| e. Add and subtract numbers? | | 5 | 4 | 3 | 2 | 1 |
| f. Concentrate? | | 5 | 4 | 3 | 2 | 1 |
| g. Think quickly? | | 5 | 4 | 3 | 2 | 1 |
| h. Solve problems? | | 5 | 4 | 3 | 2 | 1 |
| These questions are about how you feel, about changes in your mood and about your ability to control your emotions since your stroke. | | | | | | |
| 3. In the past week, how often did you … | | None of the time | A little of the time | Some of the time | Most of the time | All of the time |
| a. Feel sad? | | 5 | 4 | 3 | 2 | 1 |
| b. Feel that there is nobody you are close to? | | 5 | 4 | 3 | 2 | 1 |
| c. Feel that you are a burden to others? | | 5 | 4 | 3 | 2 | 1 |
| d. Feel that you have nothing to look forward to? | | 5 | 4 | 3 | 2 | 1 |
| e. Blame yourself for mistakes? | | 5 | 4 | 3 | 2 | 1 |
| f. Enjoy things as much as you ever have? | | 5 | 4 | 3 | 2 | 1 |
| g. Feel quite nervous? | | 5 | 4 | 3 | 2 | 1 |
| h. Feel that life is worth living? | | 5 | 4 | 3 | 2 | 1 |
| i. Smile and laugh at least once a day? | | 5 | 4 | 3 | 2 | 1 |
| The following items are about your ability to communicate with other people, as well as your ability to understand what you read and what you hear in a conversation. | | | | | | |
| 4. In the past week, how difficult was it to … | | Not difficult at all | A little difficult | Somewhat difficult | Very difficult | Extremely difficult |
| a. Say the name of someone whose face was in front of you? | | 5 | 4 | 3 | 2 | 1 |
| b. Understand what was being said to you in a conversation? | | 5 | 4 | 3 | 2 | 1 |
| c. Reply to questions? | | 5 | 4 | 3 | 2 | 1 |
| d. Correctly name objects? | | 5 | 4 | 3 | 2 | 1 |
| e. Participate in a conversation with a group of people? | | 5 | 4 | 3 | 2 | 1 |
| f. Have a conversation on the telephone? | | 5 | 4 | 3 | 2 | 1 |
| g. Call another person on the telephone (select the correct phone number and dial)? | | 5 | 4 | 3 | 2 | 1 |
| The following items ask about activities you might do during a typical day. | | | | | | |
| 5. In the past 2 weeks, how difficult was it to … | | Not difficult at all | A little difficult | Somewhat difficult | Very difficult | Cannot do at all |
| a. Cut your food with a knife and fork (Use chopsticks to hold food)? | | 5 | 4 | 3 | 2 | 1 |
| b. Dress the top part (waist up) of your body? | | 5 | 4 | 3 | 2 | 1 |
| c. Bathe yourself? | | 5 | 4 | 3 | 2 | 1 |
| d. Clip your toenails? | | 5 | 4 | 3 | 2 | 1 |
| e. Get to the toilet on time? | | 5 | 4 | 3 | 2 | 1 |
| f. Control your bladder (not have an accident)? | | 5 | 4 | 3 | 2 | 1 |
| g. Control your bowels (not have an accident)? | | 5 | 4 | 3 | 2 | 1 |
| h. Do light household tasks/chores (eg, dust, make a bed, take out garbage, do the dishes)? | | 5 | 4 | 3 | 2 | 1 |
| i. Go shopping? | | 5 | 4 | 3 | 2 | 1 |
| j. Handle money (eg, make change)? | | 5 | 4 | 3 | 2 | 1 |
| k. Manage finances (eg, pay monthly bills, manage checking account)? | | 5 | 4 | 3 | 2 | 1 |
| l. Do heavy household chores (eg, vacuum, laundry or yard work)? | | 5 | 4 | 3 | 2 | 1 |
| The following questions are about your ability to be mobile, at home and in the community. | | | | | | |
| 6. In the past 2 weeks, how difficult was it to … | | Not difficult at all | A little difficult | Somewhat difficult | Very difficult | Cannot do at all |
| a. Sit without losing your balance? | | 5 | 4 | 3 | 2 | 1 |
| b. Stand without losing your balance? | | 5 | 4 | 3 | 2 | 1 |
| c. Walk without losing your balance? | | 5 | 4 | 3 | 2 | 1 |
| d. Move from a bed to a chair? | | 5 | 4 | 3 | 2 | 1 |
| e. Get out of a chair without using your hands for support? | | 5 | 4 | 3 | 2 | 1 |
| f. Walk one block? | | 5 | 4 | 3 | 2 | 1 |
| g. Walk fast? | | 5 | 4 | 3 | 2 | 1 |
| h. Climb one flight of stairs? | | 5 | 4 | 3 | 2 | 1 |
| i. Climb several flights of stairs? | | 5 | 4 | 3 | 2 | 1 |
| j. Get in and out of a car? | | 5 | 4 | 3 | 2 | 1 |
| The following questions are about your ability to use your hand that was MOST AFFECTED by your stroke. | | | | | | |
| 7. In the past 2 weeks, how difficult was it to use your hand that was most affected by your stroke to … | | Not difficult at all | A little difficult | Somewhat difficult | Very difficult | Cannot do at all |
| a. Carry heavy objects (eg, bag of groceries)? | | 5 | 4 | 3 | 2 | 1 |
| b. Turn a doorknob? | | 5 | 4 | 3 | 2 | 1 |
| c. Open a can or jar? | | 5 | 4 | 3 | 2 | 1 |
| d. Tie a shoelace? | | 5 | 4 | 3 | 2 | 1 |
| e. Pick up a dime? | | 5 | 4 | 3 | 2 | 1 |
| The following questions are about how stroke has affected your ability to participate in the activities that you usually do, things that are meaningful to you and help you to find purpose in life. | | | | | | |
| 8. During the past 4 weeks, how much of the time have you been limited in … | | None of the time | A little of the time | Some of the time | Most of the time | All of the time |
| a. Your work, volunteer or other activities? | | 5 | 4 | 3 | 2 | 1 |
| b. Your social activities? | | 5 | 4 | 3 | 2 | 1 |
| c. Quiet recreation (crafts, reading)? | | 5 | 4 | 3 | 2 | 1 |
| d. Active recreation (sports, outings, travel)? | | 5 | 4 | 3 | 2 | 1 |
| e. Your role as a family member and/or friend? | | 5 | 4 | 3 | 2 | 1 |
| f. Your participation in spiritual or religious activities? | | 5 | 4 | 3 | 2 | 1 |
| g. Your ability to feel emotionally connected to another person? | | 5 | 4 | 3 | 2 | 1 |
| h. Your ability to control your life as you wish? | | 5 | 4 | 3 | 2 | 1 |
| i. Your ability to help others in need? | | 5 | 4 | 3 | 2 | 1 |
